# MyChemise: a 2D drawing software that uses morphing for visualisation purposes

**DOI:** 10.1186/1758-2946-4-S1-P43

**Published:** 2012-05-01

**Authors:** Jörg-Hubertus Wilhelm

**Affiliations:** 1 Sophie-Scholl-Ring 26, 31275 Lehrte, Germany

## 

2D drawing programs account for some of the first computer applications in chemistry and are widely distributed. Many publications, especially in recent time, show that this sector is developing continually [1-3] . MyChemise (My Chemical Structure Editor) is a new 2D structure editor [4]. It is designed as a Java applet that enables the direct creation of structures in the Internet using a web browser. MyChemise saves files in a digital format (.cse) and the import and export of .mol files using the appropriate connection tables is also possible.

MyChemise is available as a free online version in English and German from the author's homepage [5]. In addition to the known ways of drawing chemical structure formulas, there are also parts implemented in the program that allow the creation of different types of presentation. The morphing module uses this technology as a component for dynamic visualization. Four-point mapping (projective mapping) is mathematically solved using the unit squares method [6]. For example, it enables a clear and simple illustration of molecule vibrations and reaction sequences.
